# A deceptive MRI appearance of the medial meniscus in a 14 year old boy: a case report

**DOI:** 10.1186/1757-1626-2-16

**Published:** 2009-01-06

**Authors:** Padmanabhan Subramanian, Charles A Willis-Owen, David G Houlihan-Burne

**Affiliations:** 1 Dept Orthopaedics, Hillingdon Hospital, Pield Heath Road, Uxbridge, Middlesex UB8 3NN, UK

## Abstract

**Background:**

A 14 year old boy with a history and clinical examination suggestive of a medial meniscal tear is presented.

**Case presentation:**

The MRI findings suggested a horizontal cleavage tear so arthroscopy was carried out. No intra articular pathology was found at the time of surgery.

**Conclusion:**

The role of MRI in investigation of meniscal injuries in children is discussed and the limitations highlighted.

## Background

Arthroscopy is the gold standard for diagnosis of meniscal tears, and allows concomitant repair or partial resection. However it is important to minimize the number of 'negative arthroscopies' (where no abnormality is found) due to the potential morbidity of general anesthaesia and surgery.

The use of MRI (Magnetic Resonance Imaging) as an adjunct to clinical assessment for the evaluation of acute meniscal tears in adults is widespread. The sensitivity has been reported to range from 79–97% and the specificity from 77–92%[1,2]. The presence of high signal within the meniscus is usually abnormal and that which extends to the articular surface on more than one image is highly suggestive of a meniscal tear[3]. MRI coupled with careful clinical assessment can result in a much reduced 'negative arthroscopy rate'[4].

The accuracy of MRI for detecting meniscal tears in children is less than in adults but has not been so thoroughly studied. It is generally accepted that high signal within the meniscus represents increased vascularity, and that this decreases with age.

High signal within the meniscus has been demonstrated in 80% of asymptomatic menisci in 10 year olds, decreasing to 35% in 15 year olds[5]. One study of 51 children with suspected meniscal pathology found a sensitivity and specificity of 50% and 78% respectively for lateral meniscal tears in the under 15 age group. They found a low positive predictive value (20%), but a high negative predictive value (93–100%) of MRI for meniscal tears[6].

## Case presentation

A 13 year old Caucasian schoolboy and keen athlete was referred to the orthopaedic clinic with right knee pain. He was experiencing swelling and considerable pain localised to the medial side of the knee following exertion. He denied locking or clicking, but reported that the knee did occasionally give way. There had been no specific injury. He had no other medical problems and no relevant family history.

Examination revealed normal alignment and a full range of motion. The medial joint line was tender. All ligaments were stable. The differential diagnosis at this stage included a medial meniscal tear or osteochondritis dissecans. Plain radiographs were unremarkable. An MRI scan was arranged.

**Figure 1:**
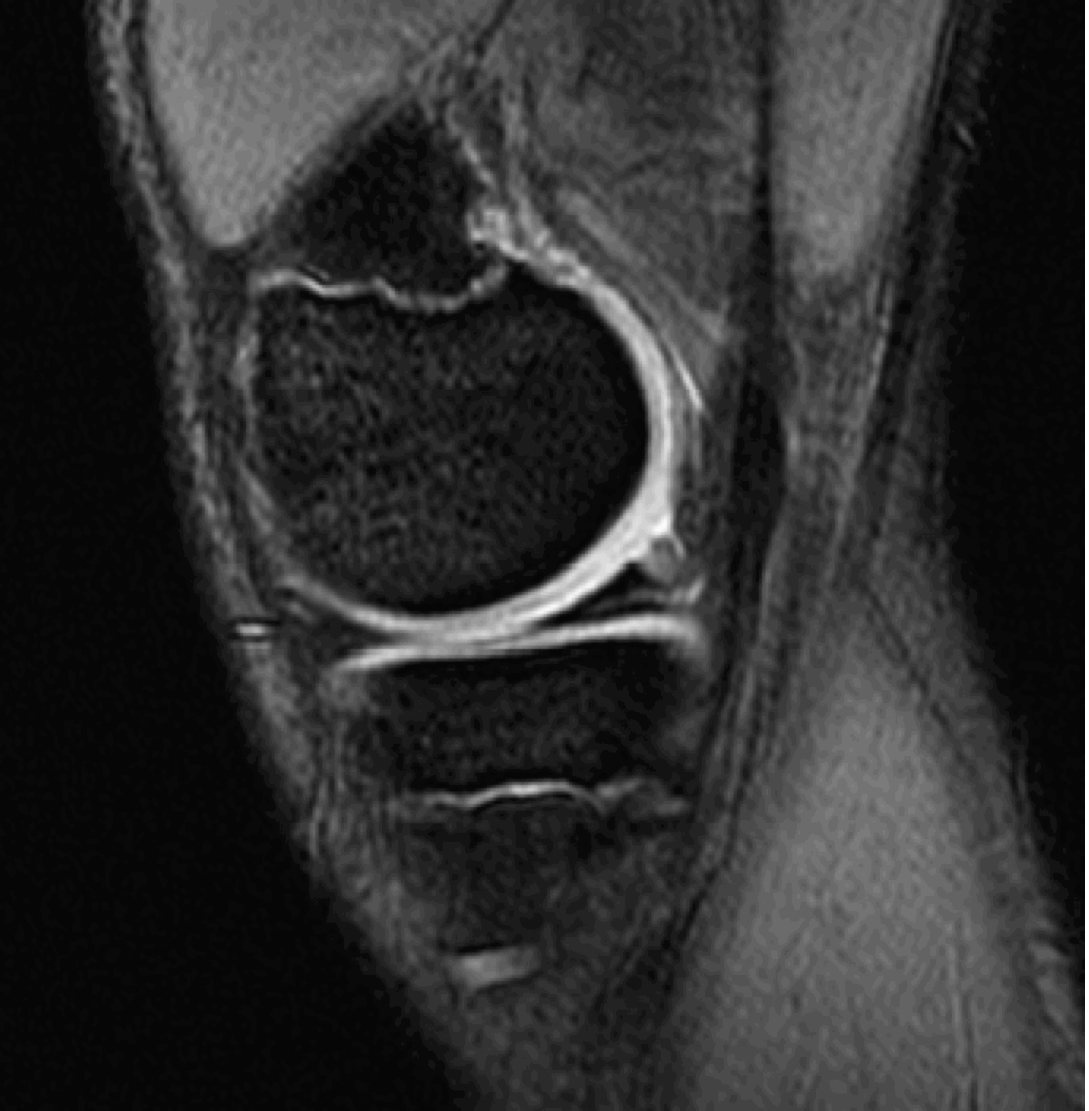
**T2 weighted Saggital MRI showing the medial meniscus**.

The MRI scan (see figure 1) showed increased signal in the posterior horn, but by this time his symptoms had settled considerably so conservative management was pursued.

One year later, during a rugby match, the patient suffered an episode of giving way associated with severe medial sided pain and a tense effusion. Following this event he had intermitted medial sided pain, clicking and subjective giving way.

Examination at this stage revealed a 5 degree extension deficit, marked medial joint line tenderness and a positive McMurrays test (clicking and pain). His anterior cruciate ligament appeared intact. On the basis of these findings and the previous MRI scan the most likely diagnosis was felt to be a medial meniscal tear and arthroscopic surgery was arranged.

**Figure 2:**
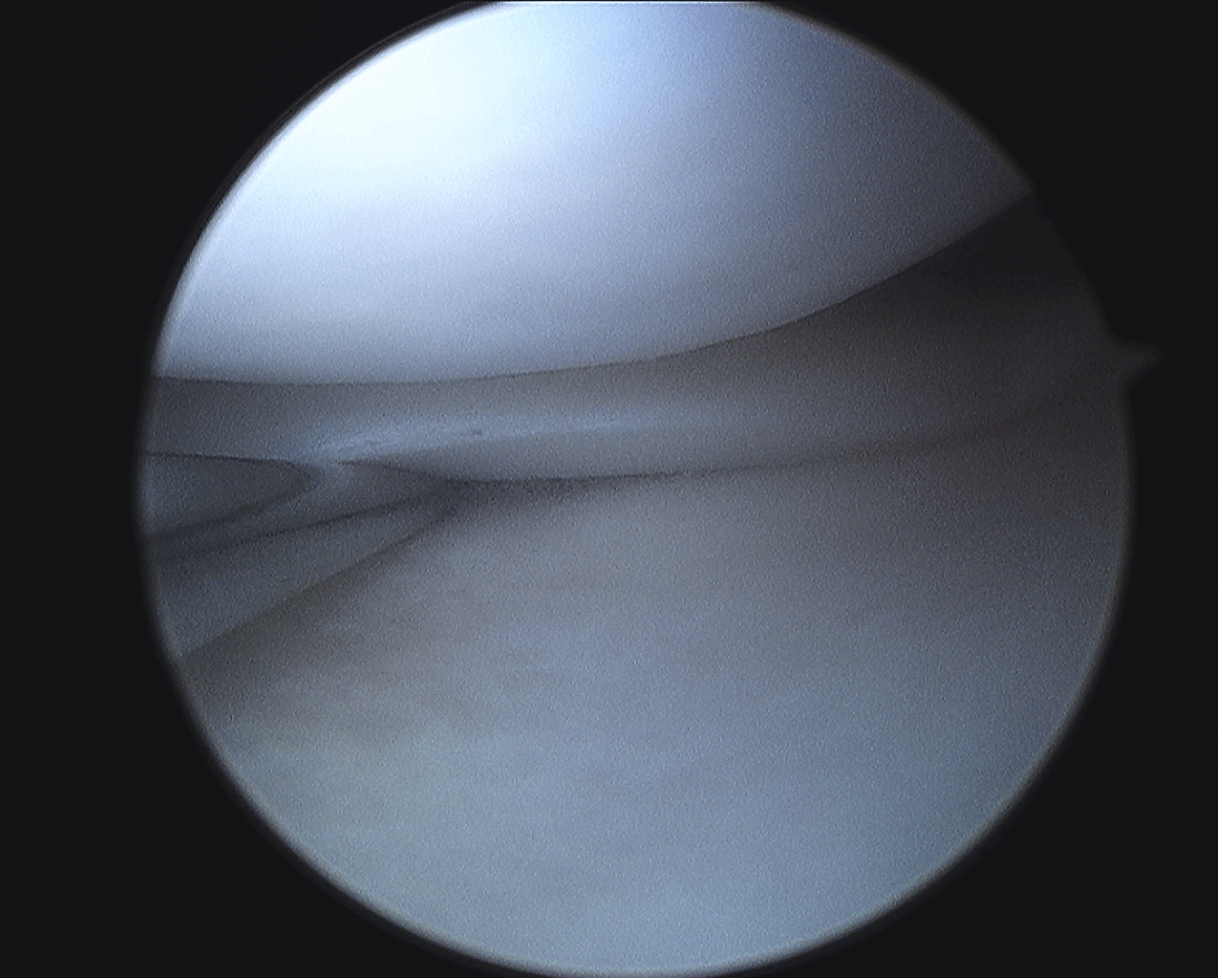
**Arthroscopic photograph showing normal medial meniscus**.

**Figure 3:**
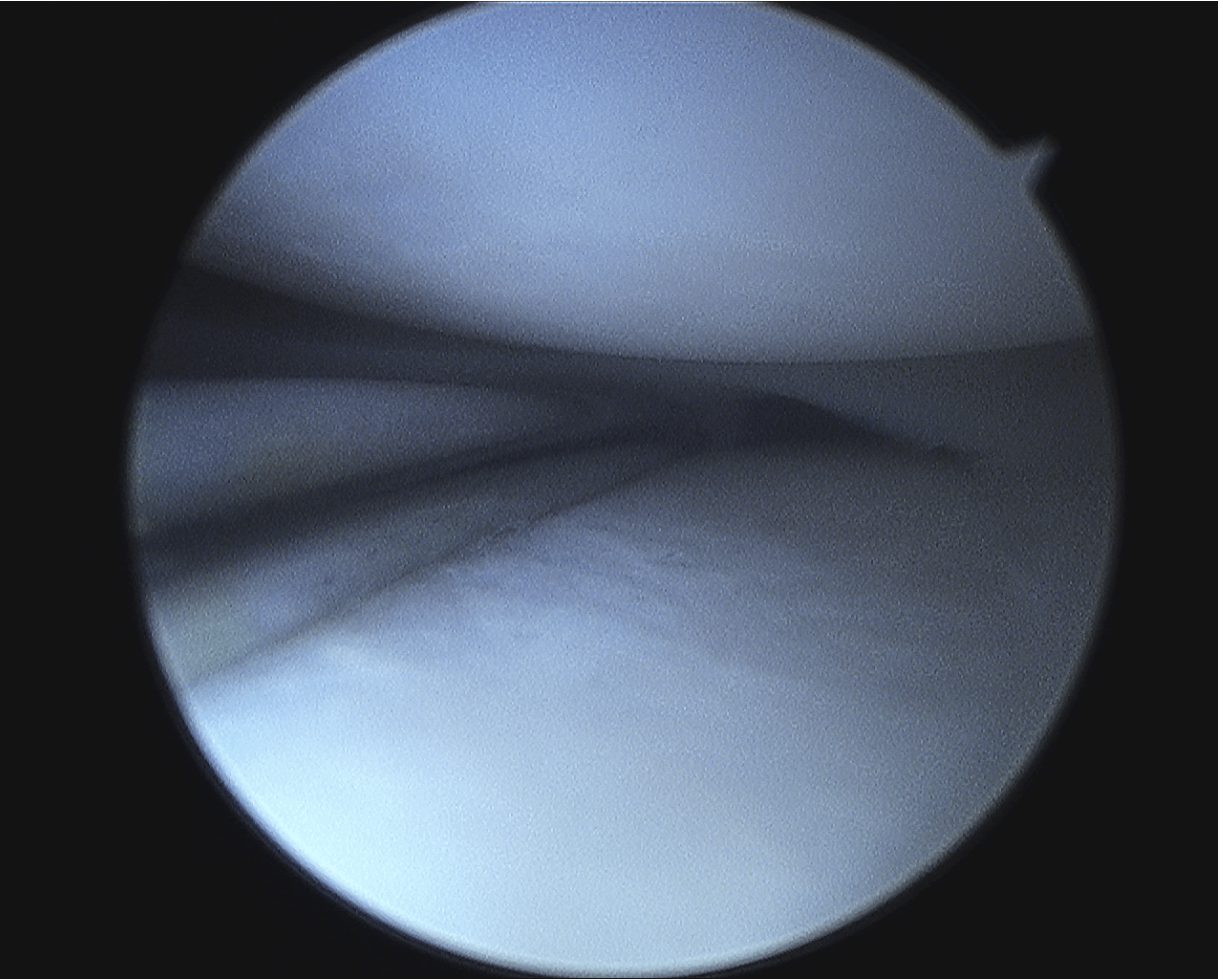
**Arthroscopic photograph showing normal medial meniscus**.

**Figure 4:**
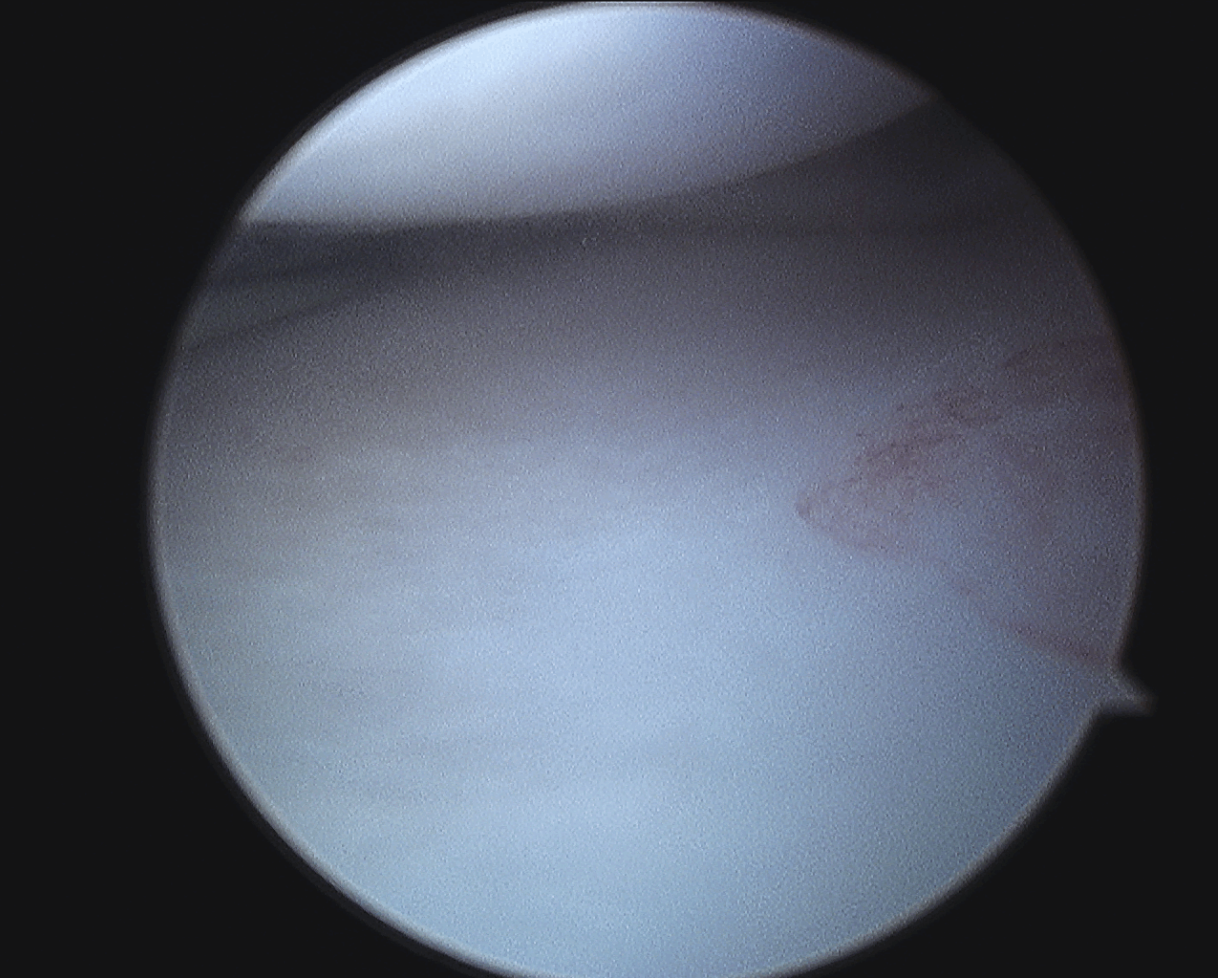
**Arthroscopic photograph showing normal lateral meniscus**.

**Figure 5:**
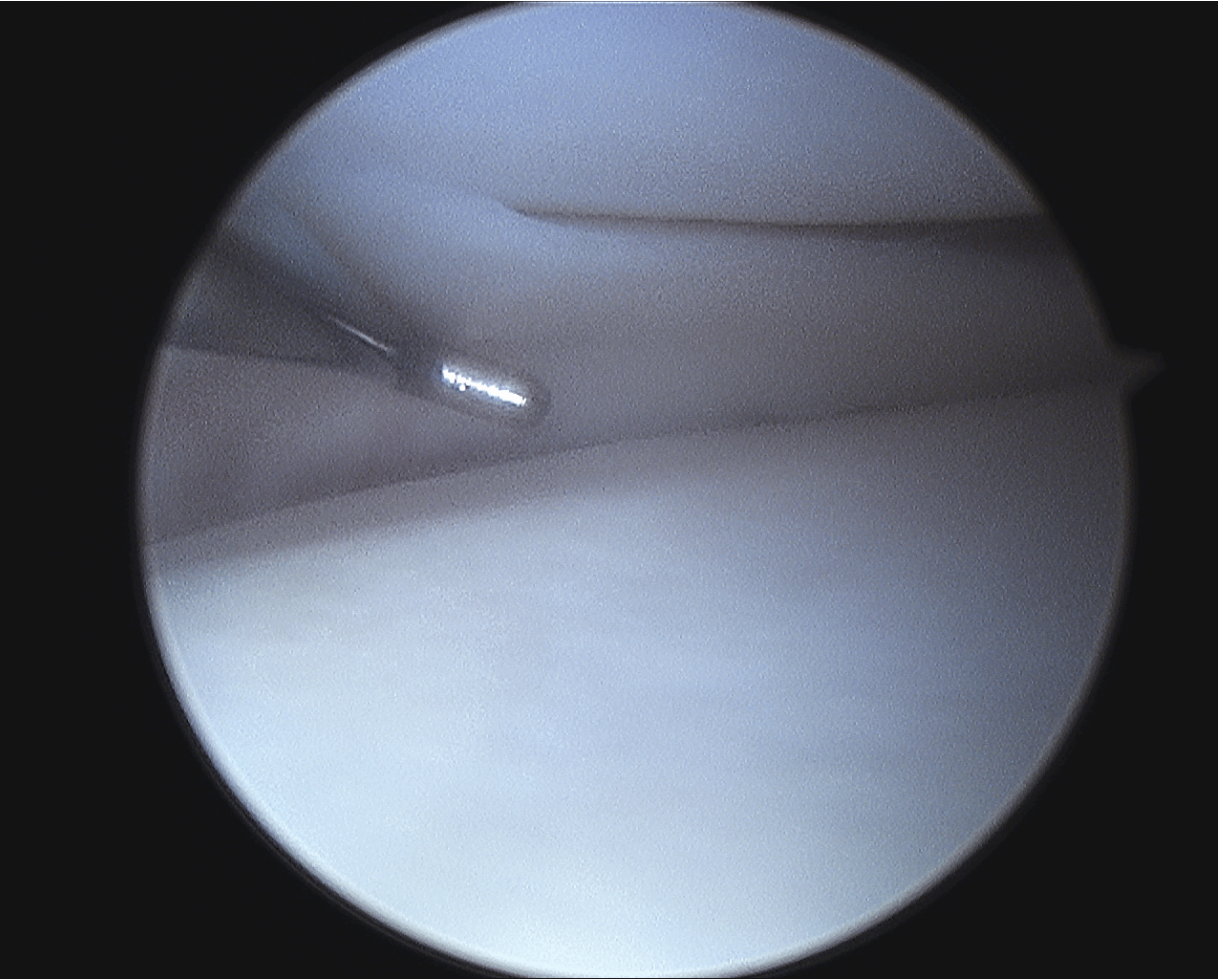
**Arthroscopic photograph showing normal lateral meniscus**.

Examination under general anaesthesia and right knee arthroscopy was carried out without complication. When anaesthetised the extension deficit was absent. Both medial and lateral menisci and all intra articular structures were normal (See figure 2, 3, 4, 5). Of particular note there was no evidence of healing or scarring of the medical meniscus. No intervention was carried out.

The patient made a prompt recovery from surgery and felt that the knee had improved. He gradually returned to sport after 2 weeks, and had not had further problems.

## Discussion

This case report aims to highlight the pitfalls associated with the interpretation of MRI scans in paediatric knee problems. Whilst the negative predictive value may be high, what appears to be a positive scan must be interpreted with caution.

The diagnostic criteria and grading of meniscal changes used in adults are less applicable in the younger age group, however there are no paediatric equivalent criteria in widespread use. Also the change in MRI appearance of the meniscus seems to be a gradual process with the adolescent meniscus taking on more adult characteristics[5]. There is probably no specific age under which these criteria become unreliable.

In adults the use of 3.0 tesla scanners and 2.0 mm slices has been shown to improve the accuracy of diagnosis of meniscal injuries (a sensitivity and specificity of 96% and 97% respectively has been reported)[7]. This technology may be of use in the paedicatric population but has not yet been thoroughly assessed.

Magnetic resonance arthrography (intra-articular injection of a Gadolinium containing solution) has been shown to significantly improve the diagnostic performance of MRI in post menisectomy patients[8]. In a comparative study both intravenous paramagnetic contrast or intra articular contrast have been shown to give similar significant increases the accuracy of MRI for detecting meniscal tears in the post partial menisectomy setting[9]. The use of contrast enhanced scanning for the diagnosis of meniscal tears in children requires further evaluation but may have a role to play in increasing diagnostic accuracy.

Ultrasound scanning has been shown to have comparable accuracy as conventional MRI in the diagnosis of meniscal tears in adults [10]. Its use for diagnosing meniscal tears in children is not clear, but the vascularity of the immature meniscus would not be expected to produce artifact resembling a tear.

## Conclusion

The use of MRI scanning in the evaluation of meniscal injuries in children is less reliable than in adults. Images must be interpreted with caution, as the sensitivity, specificity and positive predictive value are all reduced. Patients and parents should be warned of the possibility of finding intact menisci at arthroscopy.

## Consent

Written informed consent was obtained from the patient for publication of this case report and accompanying images. A copy of the written consent is available for review by the Editor-in-Chief of this journal.

## Competing interests

The authors declare that they have no competing interests.

## Authors' contributions

PS and CWO saw the patient pre and post operatively and contributed to the manuscript equally, DHB carried out the arthroscopy.
